# Effect of Dust and Anthropogenic Aerosols on Columnar Aerosol Optical Properties over Darjeeling (2200 m asl), Eastern Himalayas, India

**DOI:** 10.1371/journal.pone.0040286

**Published:** 2012-07-05

**Authors:** Abhijit Chatterjee, Sanjay K. Ghosh, Anandamay Adak, Ajay K. Singh, Panuganti C. S. Devara, Sibaji Raha

**Affiliations:** 1 Environmental Sciences Section, Bose Institute, Kolkata, India; 2 Centre for Astroparticle Physics and Space Science, Bose Institute, Darjeeling, India; 3 Centre for Astroparticle Physics and Space Science, Bose Institute, Kolkata, India; 4 Department of Physics, Bose Institute, Kolkata, India; 5 Indian Institute of Tropical Meteorology, Pune, India; University of Oxford, United Kingdom

## Abstract

**Background:**

The loading of atmospheric particulate matter (aerosol) in the eastern Himalaya is mainly regulated by the locally generated anthropogenic aerosols from the biomass burning and by the aerosols transported from the distance sources. These different types of aerosol loading not only affect the aerosol chemistry but also produce consequent signature on the radiative properties of aerosol.

**Methodology/Principal Findings:**

An extensive study has been made to study the seasonal variations in aerosol components of fine and coarse mode aerosols and black carbon along with the simultaneous measurements of aerosol optical depth on clear sky days over Darjeeling, a high altitude station (2200 masl) at eastern Himalayas during the year 2008. We observed a heavy loading of fine mode dust component (Ca^2+^) during pre-monsoon (Apr – May) which was higher by 162% than its annual mean whereas during winter (Dec – Feb), the loading of anthropogenic aerosol components mainly from biomass burning (fine mode SO_4_^2−^ and black carbon) were higher (76% for black carbon and 96% for fine mode SO_4_^2−^) from their annual means. These high increases in dust aerosols during pre-monsoon and anthropogenic aerosols during winter enhanced the aerosol optical depth by 25 and 40%, respectively. We observed that for every 1% increase in anthropogenic aerosols, AOD increased by 0.55% during winter whereas for every 1% increase in dust aerosols, AOD increased by 0.46% during pre-monsoon.

**Conclusion/Significance:**

The natural dust transport process (during pre-monsoon) plays as important a role in the radiation effects as the anthropogenic biomass burning (during winter) and their differential effects (rate of increase of the AOD with that of the aerosol concentration) are also very similar. This should be taken into account in proper modeling of the atmospheric environment over eastern Himalayas.

## Introduction

The increase in the loading of atmospheric aerosols (locally generated and long-range transported) over the Himalaya is a matter of concern, since most of the glaciers in the region have been retreating since 1850 with increasing melting rates [1]. The rising anthropogenic interferences for rapid urbanization and development in the Himalaya not only affect the immediate landscape environment, but also the atmospheric environment which is becoming an increasing concern [2]. Most of the studies on physical and chemical characterization of aerosols have been made over western and north-western Himalaya but as far as the eastern Himalaya is concerned, ours was the first study [3] on a thorough chemical characterization of aerosol at a high altitude station.

A strong seasonal variation in aerosol loading over this region had been observed and reported in our earlier study [3]. During summer and pre-monsoon, aerosols are transported from arid and semi-arid regions of India and beyond to the Himalaya driven by the westerly pre-monsoon winds. Due to enhanced convection and the steep pressure gradient across the Himalayan-Gangetic region, these aerosols are transported to the higher altitudes [4]. With the onset of summer monsoon, the heavy dust loading significantly diminishes due to aerosol washout from the atmosphere and enhances the loading of sea salt aerosols to a significant level. During post-monsoon and winter, north-easterly winds from sub-continents bring anthropogenic aerosols over the Himalayan region. In addition to that, biomass burning during winter also plays a role in loading of anthropogenic aerosols over eastern Himalayas [3]. These distinctly different seasonal behaviors of aerosol not only affect the aerosol chemistry but also produce consequent signature on the optical properties of aerosols.

One of the most common and important aerosol optical properties is aerosol optical depth (AOD) which has been used by several researchers as an aerosol loading indicator to elucidate the high pollution levels over a region. The columnar aerosol content is the resultant of the different aerosol types and would undergo seasonal/temporal changes associated with the synoptic/regional meteorology and/or source strength. This would lead to distinct spectral variation of AODs [5] and consequent impacts on the aerosol optical properties and radiative forcing [6]. The characterization of this spectral dependence through the intuitive understanding and interpretation of optical depth spectra help us in modeling the radiative effects of aerosols and the retrieval of its optical properties.

In this paper, we present the seasonal variation of columnar AODs at five different wavelengths and its dependence on the atmospheric loading of fine (aerodynamic diameter less than 2.5 µm) and coarse (aerodynamic diameter greater than 2.5 µm) mode aerosols and its components over Darjeeling, a high altitude station in eastern Himalaya, India during Jan – Dec 2008. This study is mainly focused on the heavy dust loading transported from long distances during pre-monsoon and anthropogenic aerosols generated locally due to biomass burning during winter and its impact on the columnar aerosol optical depth.

### Study Site with Prevailing Meteorology

Darjeeling, a hill station in eastern India, with a population of ∼100,000 persons, is one of the most popular tourist destinations in the entire world. The overall areas of the Darjeeling district and Darjeeling Township are about 1200 sq. km and 11.44 sq. km, respectively. Darjeeling Township is located at an average altitude of ∼2000 m above mean sea level (amsl) and surrounded by different types of topography of the lower-eastern-Himalayas. The southern region comprises the marshy low-lying area at an average height of ∼100–300 m amsl. The apex is formed by the Phalut ridge (altitude 3800 m amsl) at the border between Nepal and India. The eastern frontier lies along two rivers, locally called Tista and Rangeet. The map of the experimental site is given in our earlier paper [3].

The experiments were carried out on the terrace of a three-storied building (∼15 m above ground level) on our institute premises. This site (latitude: 27°01′N, longitude: 88°15′E with an altitude of 2194 m amsl) is at an altitude of about 200 m above the main township and is a remote area compared to the main township with a limited number of residential houses and forested areas dominated by juniper and verities of pine, in the immediate vicinity of the observatory. The closest street with significant road traffic is about 200 m away of the study site. The area, within a radius of ∼10 km is occupied by several major and minor tea processing units operated by furnace oil and coal and several tea gardens where several ammoniated fertilizers are used. Wood and biomass burning in the nearby villages, automobile exhaust (mainly tourist vehicles) throughout the year and the exhaust from the “Toy Train” (Darjeeling Himalayan Railway), which is enlisted as an UN (United Nations) world-heritage and still runs on coal as its fuel, are the major sources of air pollution at this hill station.

The average temperature during the study period was found to be 16.5±5°C with minimum of 6.8°C during January and maximum of 22.5°C during June. In general the relative humidity was high across the whole study period with an average of 84%. The dry season (Oct - May) remained moderately dry with an average relative humidity of 76% compared to the wet season (June – Sept) with an average relative humidity of 92%. The total rainfall during the entire study period (Jan-Dec) was found to be 3315 mm, 85% of which was during wet season (2820 mm). The surface wind pattern during winter was mainly easterly and northeasterly (from continental areas covering densely populated cities) with average speed of 0.92 ms^−1^ and during monsoon it was mainly southeasterly and southwesterly (from the Bay of Bengal and Arabian Sea) with an average speed of 1.32 ms^−1^. During pre-monsoon, the average wind speed was maximum (2.4 ms^−1^) and the direction was mainly from westerly and northwesterly. These distinctly different wind fields impart extreme temporal variability in aerosol characteristics.

## Methods

### 1. Aerosol Collection and Chemical Analysis

A respirable dust sampler (model APM 460BL) and a fine dust sampler (model APM 550) manufactured by Envirotech Instrument Pvt Ltd, India were used for the collection of PM_10_ (aerodynamic diameter less than 10 µm) and PM_2.5_ (aerodynamic diameter less than 2.5 µm) aerosol respectively. The technical specifications of the aerosol sampler along with their working principle and the chemical analysis of aerosol components (water soluble ionic species) has been described in detail in Chatterjee et al. [3].

### 2. Black Carbon (BC) Measurements

Black carbon aerosol measurements have been carried out using Aethalometer (Model AE-21) of Magee Scientific, USA. The Aethalometer makes measurements of mass concentration of aerosol black carbon by measuring the attenuation of light transmitted through a quartz filter tape on to which the ambient particles are made to impinge. The attenuation of the intensity (*I )* transmitted through the collecting part of the filter relative to the intensity (*I*_0_ *)* through the reference part is *A*  = 100 ln (*I*_0_ */I )* and is proportional to the surface concentration of black carbon. More details are available elsewhere (http://www.mageesci.com/Aethalometer_abook_2009.pdf).

### 3. Measurements of Columnar AOD

The spectral AOD was measured at five wavelengths centered on 380, 500, 675, 936 and 1020 nm using a handheld multichannel Sun photometer (Microtops-II). The full width at half maximum (FWHM) bandwidth at each of these wavelength channels is 2.4±0.4 nm, and the accuracy of the Sun-targeting angle, i.e., field of view (FOV), is ∼ 2.5°. To avoid any error in sun targeting the Microtops-II was mounted on a tripod stand. AOD was measured at ∼ 30-min intervals during day time on clear-sky conditions over our institute campus where the aerosol sampling was also done.

#### 3.1. Estimation of Angstrom exponent from AOD

The wavelength dependence of AOD is characterized by the Angstrom wavelength exponent α which also provides some basic information on the aerosol size distribution. When AODs are estimated at a number of wavelengths, a linear regression analysis is performed using Eq.(1) in a log–log scale and the slope of the regression line is taken as α.
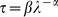
(1)


where τ is the AOD at the wavelength λ expressed in micrometer (µm) and β, the Angstrom turbidity coefficient (and is numerically equal to the AOD at 1 µm). This representation is fairly accurate over short ranges of wavelength. However, the application of Eq. (1) over a wide wavelength range would lead to significant inaccuracies, particularly when multimodal aerosol size distribution exists in the vertical atmospheric column over the study region [7], [8]. These modes would represent aerosols of different types, from distinctively different sources such as industrial, urban, biomass burning, transported dust or sea-salt. Under such conditions it is imperative for the Angstrom exponent α, to deviate from the simple monotonic λ dependence given by Eq. (1) and show curvature in the λ and τ space [7,8,9, and 10]. In such cases, the derivative of α (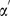) can be used to provide information on aerosol size distribution. 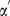
is numerically estimated using the following approximation [11].
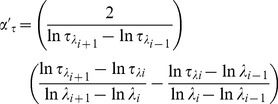
(2)


where λ_i-1,_ λ_i_ and λ_i+1_ are the discrete wavelengths at 0.4, 0.5 and 1.02 µm (either end of the wavelength range and a near mid point) and τ_λi-1_, τ_λi_ and τ_λi+1_ are the corresponding AODs. Similarly 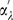
can be determined as follows
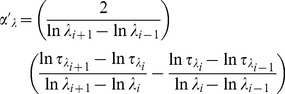
(3)


α greater than 1.0 (and the positive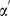) indicate the dominance of fine mode aerosols over coarse mode whereas α less than 1.0 (and negative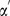) indicate the dominance of coarse mode aerosols over fine mode.

### 4. Meteorological Parameters

The hourly meteorological data for all the sampling days during the entire study period were obtained from India Meteorological Department, Govt. of India. This meteorological station is situated at a place called “Raj Bhavan” in Darjeeling which has the same altitude and the same environment with our institute campus/observational site and which is around 300 m away from our institute campus/observational site.

### 5. Data Sets

The aerosol samples were collected during the entire study period (Jan-Dec) in 2008. A total of 120 samples were collected (86 during dry season and 34 during wet season) for each of PM_2.5_ and PM_10_. Each sampling was started at 0330 hrs (UTC) and run for ∼24 hrs. Both the samplers were placed on the terrace of a three-storied building (∼15 m above ground level) on our institute premises. PM_2.5_ and PM_10_ aerosol samples were collected simultaneously on same days and for the same durations. Depending on the availability of the clear-sky days, AOD measurements were carried out for 79 days (throughout the day) during the entire study period (66 days during dry season and 13 days during wet season). The data of black carbon was obtained from Aethalometer at every 5-min interval through-out the study period.

## Results and Discussion

### 1. Temporal Variation of AOD and Angstrom Exponent: Effect of Total Aerosol Loading

**Figure 1:**
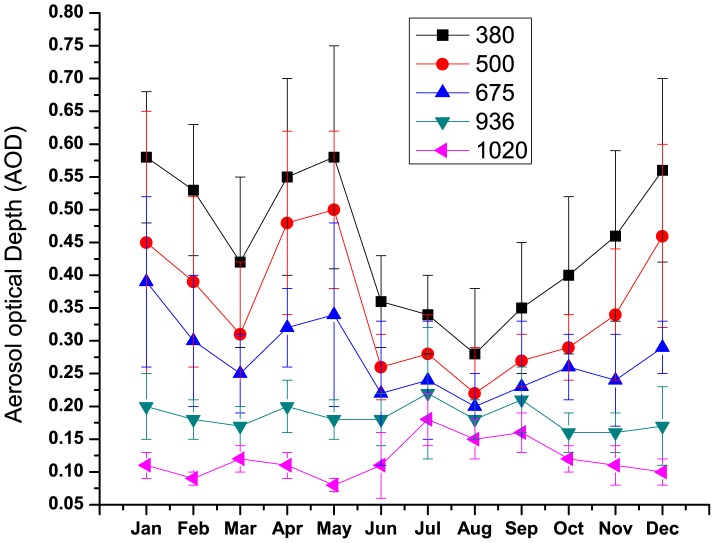
Monthly mean columnar AODs at five different wavelengths (nm).

**Figure 2:**
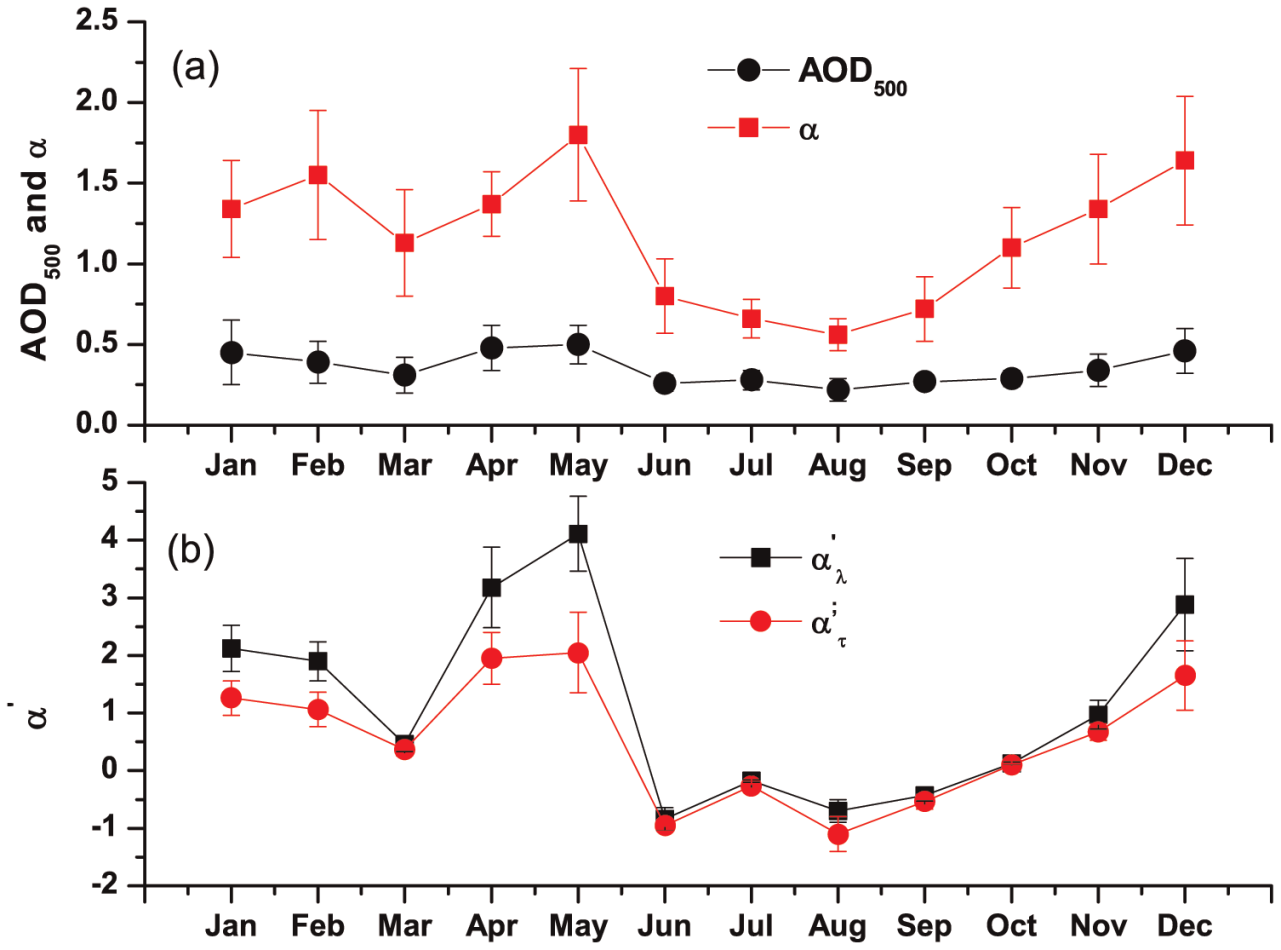
Seasonal variations in a) AOD at 500 nm and Angstrom exponent and b) derivative of Angstrom exponent.

**Figure 3:**
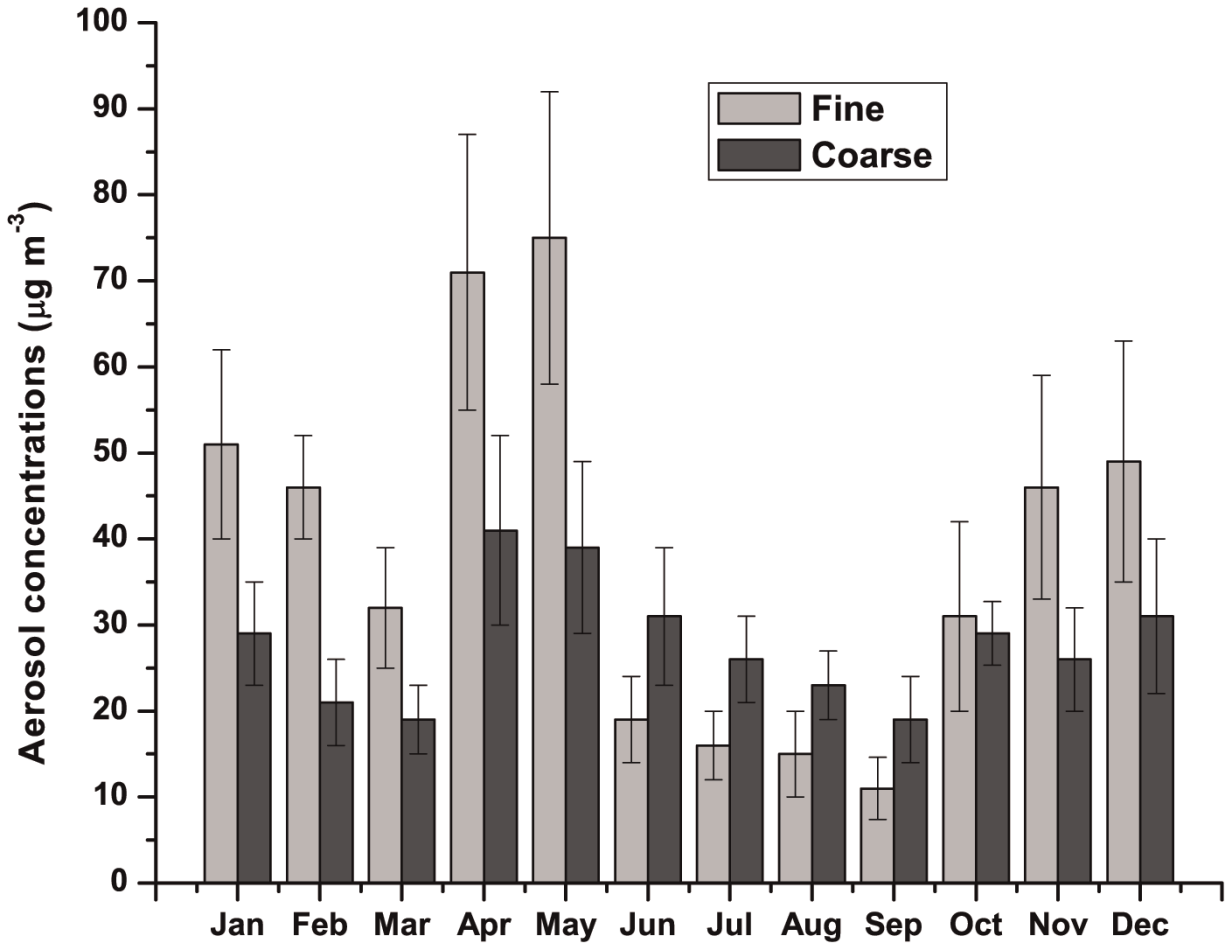
Seasonal variations in fine and coarse mode aerosol concentrations.

The monthly mean columnar AODs at five different wavelengths during the entire study period are shown in Figure 1. Here we observe two distinct features in AOD spectra between dry (Oct – May) and wet (Jun – Sep) seasons. During dry season, AOD was found to decrease rapidly whereas during wet season, AOD decreased slowly as wavelength increased. Also, there was a small increase in AOD at higher wavelength during wet season compared to dry season. The temporal variations in AOD at 500 nm (AOD_500_) and α are shown in Figure 2(a). It clearly shows that both AOD_500_ and α were consistently higher during January to May, remained low during June to September and started increasing from October till December. The temporal variations of α′_λ_ and α′_τ_ are shown in Figure 2(b). α′_λ_ and α′_τ_ both showed strong seasonal variations with their positive values during dry season and negative values during wet season. While the Angstrom exponent (α) is a mere indicator of the aerosol size distribution, its derivatives impart a clearer picture of the possible types of aerosols and in turn potential sources also [12]. The positive α′ indicates the dominance of fine mode aerosol during dry season (mainly of anthropogenic, biomass burning etc) whereas negative α′ indicates the dominance of coarse mode aerosol during wet season (mainly sea-salt aerosol). Although α′_λ_ and α′_τ_ were found to be strongly correlated with each other (R^2^ = 0.95) and showed similar variations, their absolute magnitudes differed from each other. Figure 3 shows the temporal variations of fine and coarse mode aerosols during the study period_._ The variation in fine mode aerosol was similar in nature to the variation in α with the higher values during dry season and lower during wet season. It is clear from the figure that fine mode aerosol dominated over coarse mode aerosol during dry season (with positive α′) whereas the reverse was found during wet season with the relative dominance of coarse mode over fine mode aerosol (with negative α′). The prevailing airmass of continental nature during winter (Dec – Feb) brings anthropogenically dominated continental aerosols from other inland regions of India which are mainly accumulated in fine mode. Also, in winter the persistent thermal inversion and fog situations at ground level cause a considerable amount of aerosol to accumulate in the lower layer of the atmosphere. But aerosol concentration during winter is largely and mostly affected due to the biomass burning in Darjeeling [3] which mainly produces fine mode aerosols. This leads to high AOD_500_ and α along with positive α′ during winter. This might be the reason for the rapid fall of AODs with the increase in wavelengths. In contrast, during wet season the marine airmass mainly from the Bay of Bengal laden with moisture and sea-salt aerosols enhances the concentration of coarse mode aerosols over Darjeeling [3]. The sharp fall in fine mode aerosols due to wash-out effect during monsoon resulted in the sharp fall in AOD_500_ and α (with negative α′). In contrast, the decrease in the coarse mode aerosol was not significant due to the advection of coarse mode sea-salt aerosol which could lead to a weak increase in AOD at longer wavelengths. Thus the AODs were found to fall slowly with the increase in wavelengths during monsoon. Several studies [12] made earlier have shown such changes in AODs and α′ with the changes in the types of air masses. During inter-monsoon seasons (pre-monsoon and post-monsoon), the changes in the wind direction and type of air masses affect the chemical composition of aerosol. During post-monsoon (Oct –Nov), the winds change its direction from south-westerly (and south-easterly) to north-easterly which start bringing continental aerosols mainly in fine mode. The changes in α′ from negative during September to positive close to zero values during October are clearly seen in Figure 2. The changes in α′ from positive to negative during transition from pre-monsoon to monsoon and from negative to positive during transition from monsoon to post-monsoon was observed by Beegum et al. [12]. Also, they reported the values of α′ during April and May (pre-monsoon) and October (post-monsoon) which are negative but very close to zero. But the interesting observation in our study is the sudden jump in α′ from March to April. The wind changes its direction from north-easterly during winter to north-westerly and westerly during pre-monsoon. The lands become dry and solar heating increases which make atmospheric conditions conductive for picking up of dust from arid and semi-arid regions of western India as well as west Asia. This transported mineral dust is mainly accumulated in fine mode [13], resulting in the increase of fine mode aerosol concentration (Figure 3). α′ also sharply changes from low positive during March to very high positive values during April and May. But this is not reflected in α which is found to increase slightly from March to April.

Gautam et al. [14] measured AOD over several sites in India and Nepal during premonsoon (Apr – Jun) in the year 2009. They measured AOD at Jaipur (450 m asl), a near-desert region in western India and AOD was found to be 0.46, which is almost equal to that measured by this study over Darjeeling (0.48 in April and 0.5 in May). They measured AOD at Shimla (2100 m asl), a western Himalayan slope location of almost equal altitude to Darjeeling and it was found to be lower (0.33) compared to our study. Gautam et al. [14] also measured AOD over several sites at Indo-Gangetic Plains (IGP) and AOD values over those regions were found to be higher (0.60–0.64) than Darjeeling. They also measured AOD over Nepal Himalayan regions like Hetauda (465 m asl), Dhulikhel (1500 m asl) and Langtang (3670 m asl). Hetauda and Dhulikhel showed much higher AOD (0.75 and 0.73 respectively) compared to Darjeeling whereas Langtang, being a remote high altitude station (much higher than Darjeeling), showed comparatively less AOD of 0.35.

### 2. Profile of Dust and Anthropogenic Aerosol

The transport of dust aerosols from arid and semi-arid regions of India including Thar Desert and even from west Asia driven by the pre-monsoon westerlies, not only influence the plains, but due to enhanced convection, aerosols are vertically advected to the higher altitudes against the foothills of the Himalayas [15]. During pre-monsoon, enhanced convection and steep pressure gradient allow dust-rich aerosols to move upward along the slopes of the Himalayas to reach high elevated stations [15]. Carrico et al. [16] showed the long range transport of dust aerosols from west Asia reaching over Nepal Himalayan regions and they found similar concentrations in Ca^2+^ at two different altitudes 800 m and 3920 m asl. Thompson et al. [17] also reported the increase in dust loading and its effect on the reductions in monsoonal intensity from a high-resolution ice core record over Tibetan Himalaya. Singh et al. [18] studied the spectral behavior of AOD over Kanpur, an urban-industrial city in the Ganga basin in India during January 2001 to December 2003. They observed a high increase in AOD during premonsoon (Apr – May) and also in winter (Dec – Feb). The main conclusion of the study made by Singh et al. [18] was that the dust was the major contributor to aerosol optical depth during premonsoon whereas anthropogenic urban aerosols contributed during winter. These results are similar to the results found by the present study over Darjeeling. Our earlier study [3] also identified two distinct source regions; Thar deserts and Arabian deserts for the long range transport of dust aerosols reaching over Darjeeling during pre-monsoon. It was found that 45% dust loading was from Thar deserts and 32% was from Arabian deserts during pre-monsoon.

**Figure 4:**
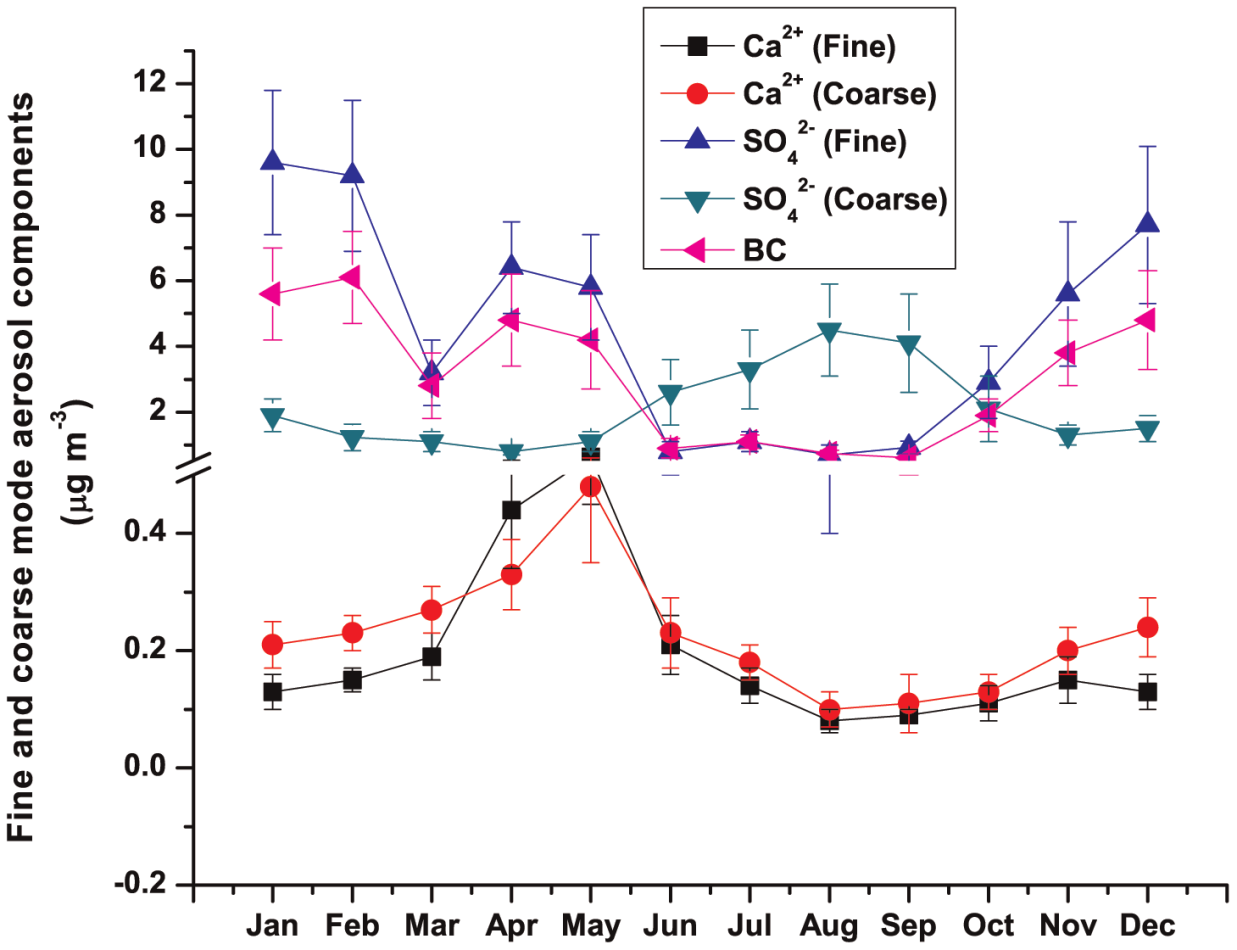
Seasonal variations in concentrations of several aerosol components.

In the present study, we have considered fine mode Ca^2+^ as the tracer of dust aerosols (mainly transported) whereas fine mode SO_4_^2−^ and BC have been considered for the tracer of anthropogenic aerosols mainly biomass burning. Figure 4 shows the seasonal variations of Ca^2+^ and SO_4_^2−^ both in fine and coarse mode and BC concentrations. The fine mode Ca^2+^ ranged between 0.08 and 0.63 µg m^−3^ whereas coarse mode Ca^2+^ ranged between 0.1 and 0.48 µg m^−3^. The annual average concentrations of Ca^2+^ in fine (0.21±0.16 µg m^−3^) and coarse mode (0.22±0.11 µg m^−3^) were found to be equal. But during pre-monsoon, fine mode Ca^2+^ showed much higher loading (0.55±0.23 µg m^−3^) compared to coarse mode (0.40±0.21 µg m^−3^). Earlier study made by Chatterjee et al. [3] also found higher concentrations of Ca^2+^ over Darjeeling during pre-monsoon in 2006. Thus the enhancement of dust-rich elements over Himalayas during pre-monsoon, which has been found to be consistent over the years, enables us to consider the fact of long-range transport of dust aerosols over Himalayas during pre-monsoon as the climatological features.

**Figure 5:**
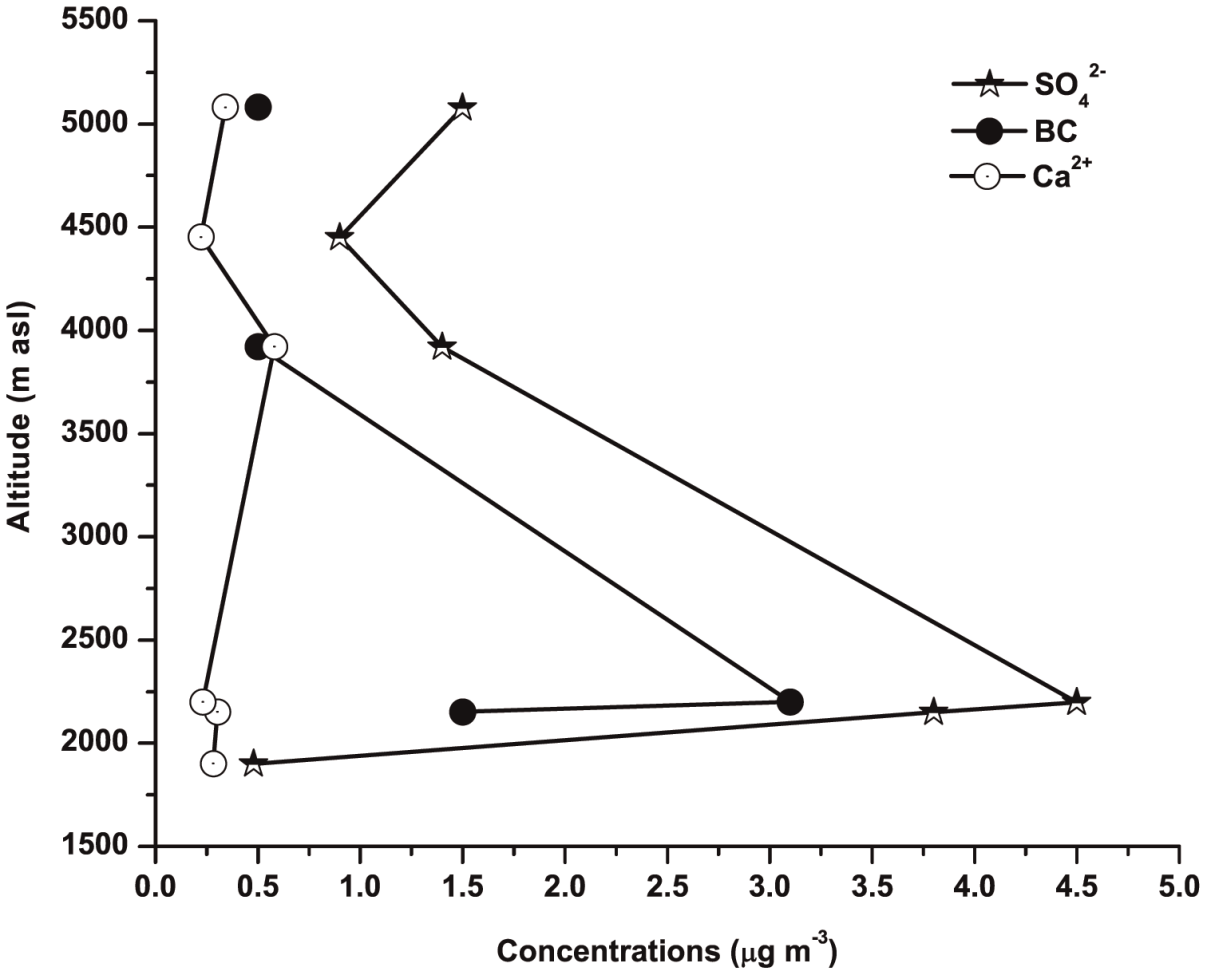
Comparisons of aerosol components between Darjeeling (2200 m) and other Himalayan sites. (Jiri, Nepal at 1900 m; Nagarkot, Nepal at 2150 m; Langtang, Nepal at 3920 m; Phortse, Nepal at 4450 m; NCO-P, Nepal at 5079 m). Data have been taken from Decesari et al. [20]. It is seen that dust component (Ca^2+^) over Darjeeling does not significantly differ from other locations during premonsoon. This vertical homogeneity of dust aerosol could be attributed to a common transport mechanism i.e. long-range transport from distant sources. Black Carbon and Sulphate aerosols over Darjeeling show significant differences from other locations. This vertical inhomogeneity could be attributed to the local anthropogenic sources.

Figure 5 shows the concentrations of Ca^2+^, SO_4_^2−^ and BC in aerosols at different locations of different altitudes over Indian and Nepal Himalayas. It is clearly seen from the figure that the Ca^2+^ concentration over Darjeeling did not significantly differ from that of other locations during pre-monsoon. Therefore Ca^2+^ seems to be homogeneously distributed vertically over the Himalayan regions. Thus there must be some common transport mechanism which brings dust aerosols to Himalayas resulting to a vertical homogeneity and this could be attributed to the long range transport of dust aerosols from arid and semi-arid regions of western India and beyond.

On the other hand, black carbon and sulphate aerosols over Darjeeling showed large deviations in their concentrations from those of other Himalayan regions during pre-monsoon. The annual average concentrations of SO_4_^2−^ in fine and coarse mode aerosols were found to be 4.5±3.3 µg m^−3^ and 2.1±1.2 µg m^−3^ respectively and thus they were higher than those of other Himalayan regions. Fine mode SO_4_^2−^ showed bimodal distribution (Figure 4) with two prominent peaks, one in winter and other in pre-monsoon. The wintertime SO_4_^2−^ aerosol was due to the biomass burning in Darjeeling whereas during pre-monsoon SO_4_^2−^ was enriched due to both the fossil fuel and biomass burning. It is to be mentioned here that pre-monsoon is the peak tourist season over Darjeeling with high influx of tourist vehicles. In contrast, coarse mode SO_4_^2−^ shows unimodal distribution with a prominent peak during monsoon. This could be attributed to the advection of larger sea-salt particles enriched with sea-SO_4_^2−^ driven by south-easterlies (or south-westerlies) marine air masses from the Bay of Bengal (or the Arabian Sea). The annual variation of BC (Figure 4) was similar in nature to that of fine mode SO_4_^2−^. The wintertime and pre-monsoon BC are attributed to biomass and fossil fuel burning respectively. BC was found to range between 0.6 and 6.1 µg m^−3^ with the average of 3.11±1.02 µg m^−3^. This was also 2–6 times higher than other locations as shown in Figure 5. However during pre-monsoon, the deviations of BC and fine mode SO_4_^2−^ concentrations over Darjeeling from other locations are due to the differences in local anthropogenic source emissions resulting vertical inhomogeneity.

### 3. Aerosol Index (AI): Transport of Dust Aerosol

**Figure 6:**
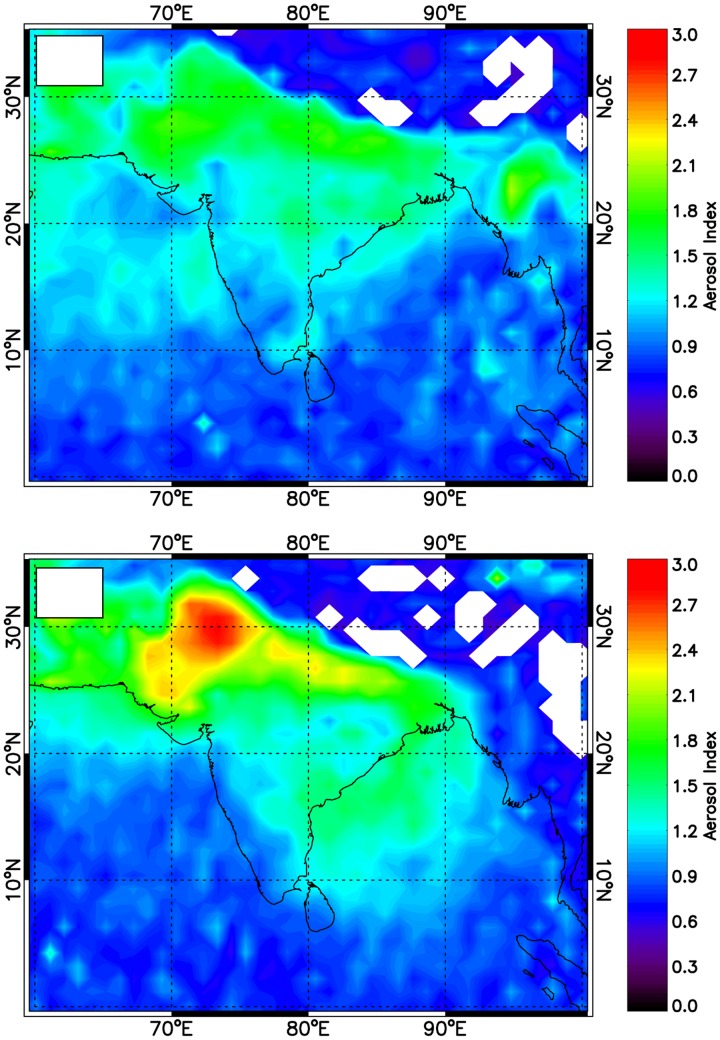
Contour plots of Aerosol Index (AI) showing dust transport over Himalaya during a) April 2008 and b) May 2008.

Aerosol solar extinction from TOMS is a valuable indicator of the total columnar aerosol loading. To better understand the high aerosol loading over eastern Himalayas, we analyzed TOMS aerosol index (AI) data during pre-monsoon (April and May) in 2008. The monthly mean AI for April and May for dust laden regions in India is shown in Figure 6. It shows the higher AI over Darjeeling and other eastern Himalayan regions during May compared to April due to strong wind-blown dust aerosol driven by westerlies. The high loading of dust aerosol over Thar deserts and its transport to Gangetic-Himalayan region through Indo-Gangetic Plain (IGP) is clearly seen from the figure. Although the AI over eastern Himalayas and eastern IGP are comparatively lower than western IGP and Thar deserts. The increasing trend of aerosol loading over IGP has been indicated by earlier studies using TOMS satellite measurements [19]. We did not study the TOMS aerosol index during winter as aerosol index depends on aerosol vertical distribution and not on surface-based aerosols and during winter, aerosols are trapped within the boundary layer under the stable atmospheric conditions.

### 4. Net Changes in AOD: Combined Effect

**Figure 7:**
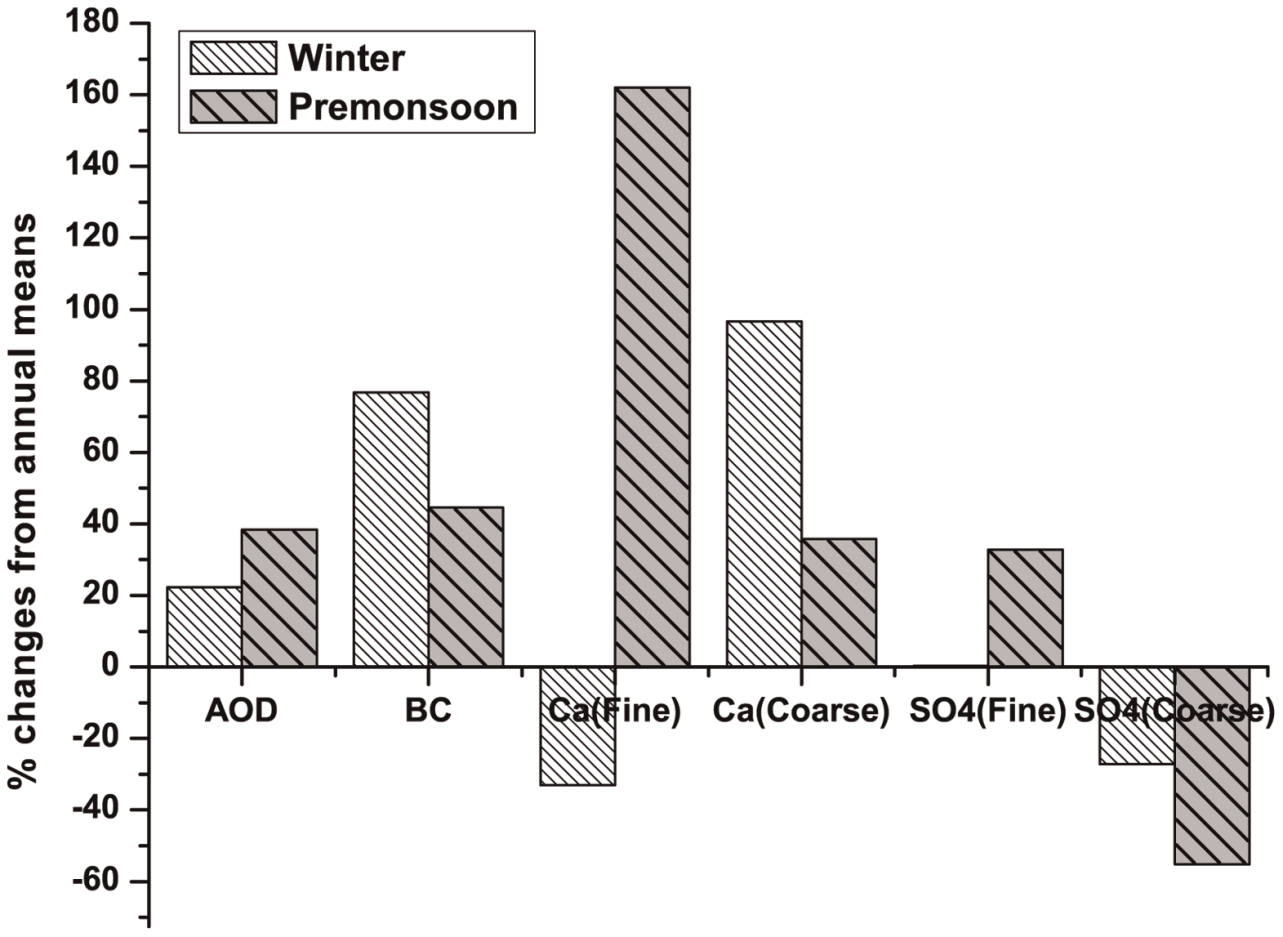
Percentage changes in aerosol component concentrations and AOD (at 500 nm) from respective annual means.

Fig. 7 shows the percentage changes in AOD_500_ during pre-monsoon and winter from the annual mean AOD. The percentage changes in the concentrations of black carbon (BC), fine and coarse mode SO_4_^2−^ and Ca^2+^ during winter and pre-monsoon from their respective annual means are also shown in this figure. It was found that AOD increased by about 25% during winter and 40% during pre-monsoon from its annual mean. The high increase in black carbon (76%) and fine mode SO_4_^2−^ (96%) concentrations could be the key factors for the enhancement of AOD during winter. These high increases in BC and SO_4_^2−^ concentrations could be attributed to the biomass burning in and around Darjeeling during winter. The concentrations of BC and fine mode SO_4_^2−^ were also higher by 45% and 36% respectively during pre-monsoon which is due to the combined effect of biomass and fossil-fuel (because of high influx of tourist vehicles as discussed earlier) burning. But the most important aspect is very high increase in fine mode Ca^2+^ (162%) during pre-monsoon which is attributed to the long range transport of dust aerosols. The increase in coarse mode Ca^2+^ concentration (32%) is five times lower comparative to fine mode Ca^2+^. Coarse mode SO_4_^2−^ on the other hand, was found to be decreased, both during pre-monsoon and winter, from its annual mean, indicating its contribution mostly from marine sources.

**Figure 8:**
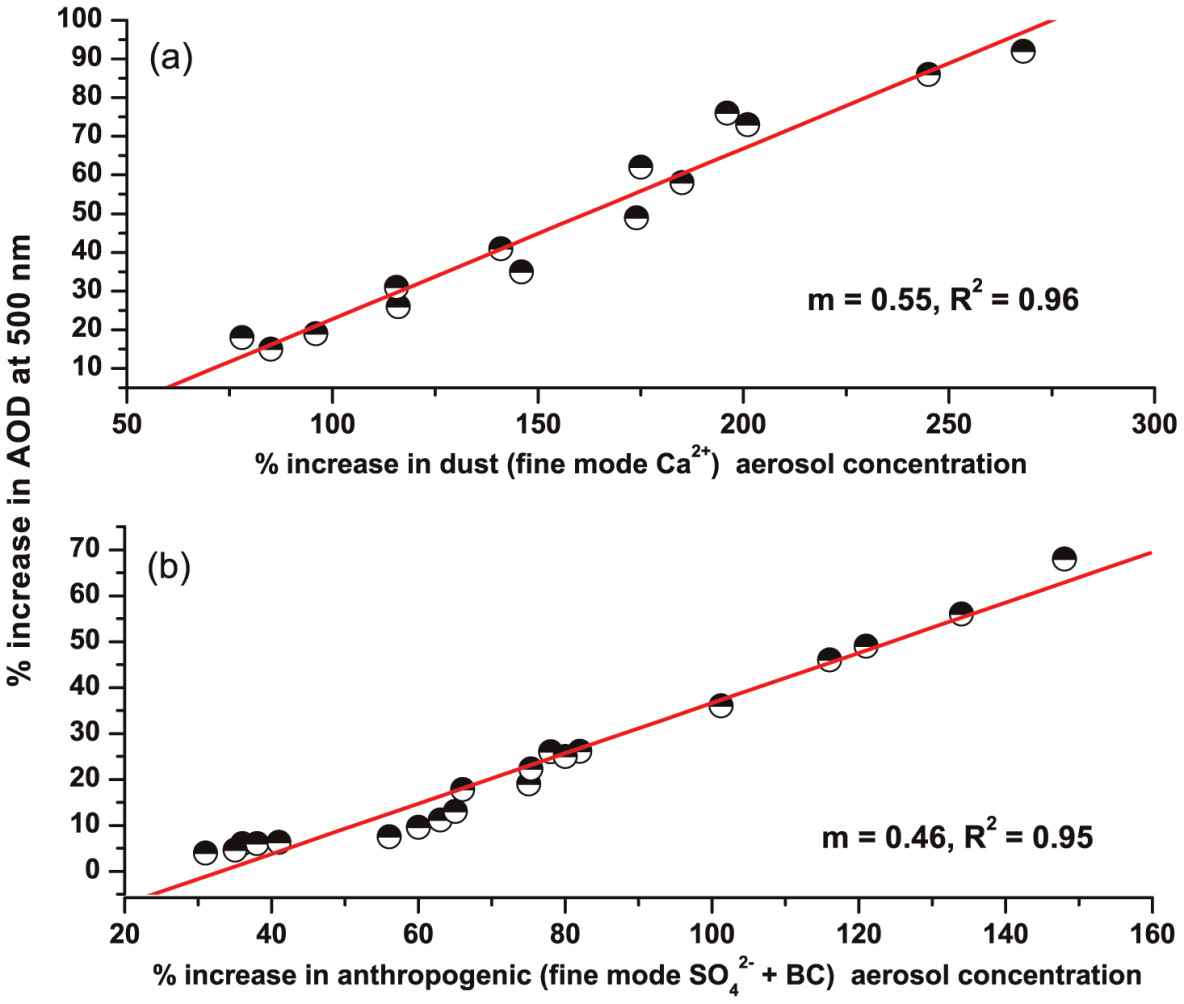
Correlations between increase in AOD (500 nm) and a) increase in dust loading during pre-monsoon and b) increase in anthropogenic aerosol loading during winter.

A graph has been plotted to show the correlations of the increase (%) in AOD_500_ with the increase (%) in total anthropogenic aerosols (BC + fine mode SO_4_^2−^) during winter (Figure 8a) and the increase (%) in dust aerosols (fine mode Ca^2+^) during pre-monsoon (Figure 8b). Here the increases in AOD and aerosol components are the increase from the respective annual means. It is clear from the figure that AOD increase shows high correlations with both the increase in anthropogenic aerosols (R^2^ = 0.96) and dust aerosols (R^2^ = 0.95). The slopes of the two linear fits show that for every 1% increase in anthropogenic aerosols, AOD increased by 0.55% during winter whereas for every 1% increase in dust aerosols, AOD increased by 0.46% during pre-monsoon.

### 5. Conclusions

The study shows a heavy loading in fine dust aerosols transported from distant sources during pre-monsoon and locally generated anthropogenic aerosols from biomass burning during winter. The higher enrichment of those aerosol components during pre-monsoon (162% increase in fine mode Ca^2+^) and winter (76% increase in black carbon and 96% increase in fine mode SO_4_^2−^) were found to be significantly higher than their annual mean concentrations which in turn affected columnar aerosol optical properties. On average, AOD was found to be increased by 25% and 40% from its annual mean during winter and pre-monsoon, respectively. It was observed that for every 1% increase in anthropogenic aerosols, AOD increased by 0.55% during winter whereas for every 1% increase in dust aerosols, AOD increased by 0.46% during pre-monsoon. It thus appears that the natural dust transport process (during pre-monsoon) plays as important a role in AOD as the anthropogenic biomass burning (during winter) and their differential effects (rate of increase of the AOD with that of the aerosol concentration) are also very similar. This should be taken into account in proper modeling of the atmospheric environment over eastern Himalayas.

Thus this study is intended to provide data on climatological aspects of aerosol loading over an urban environment in eastern Himalaya based on the ground measurements of aerosol optical depth, the single most important parameter for evaluating direct radiative forcing. This study will not only be helpful for calibration and validation of AOD retrievals from satellites but also for other satellite retrieved aerosol products. A year-long study on both chemical and optical properties of aerosol will help in evolving climatological models for radiation budget studies over Himalaya. This study will also help in proper understanding of the local atmospheric radiative balance over Himalaya. The transport of dust aerosols from distant sources over Himalaya during premonsoon and high loading of local anthropogenic aerosols from biomass burning significantly trap the downward short wave solar radiation which in turn increases the atmospheric heating rate by several factors. This could also have the significant effect on monsoon circulation by modifying the cloud-drop size distribution and affecting temperature. This may have significant implications to the water reservoirs and the regional hydrological cycle over a high-altitude eastern Himalayan region where water stress is one of the serious issues at the current scenario. Thus this study is an assessment of the effect of natural and anthropogenic aerosols on an important aerosol optical property, aerosol optical depth, may have significant climatic implications for Darjeeling, a region over eastern Himalaya which is topographically very fragile and ecologically very delicate.
